# Carcinoma and Sarcoma Microenvironment at a Glance: Where We Are

**DOI:** 10.3389/fonc.2020.00076

**Published:** 2020-03-03

**Authors:** Mattia Saggioro, Edoardo D'Angelo, Gianni Bisogno, Marco Agostini, Michela Pozzobon

**Affiliations:** ^1^Stem Cells and Regenerative Medicine Lab, Fondazione Istituto di Ricerca Pediatrica Città Della Speranza, Padova, Italy; ^2^Department of Women and Children Health, University of Padova, Padova, Italy; ^3^First Surgical Clinic, Department of Surgery, Oncology and Gastroenterology, University of Padova, Padova, Italy; ^4^NanoInspired Biomedicine Lab, Fondazione Istituto di Ricerca Pediatrica Città della Speranza, Padova, Italy; ^5^LIFELAB Program, Consorzio per la Ricerca Sanitaria-CORIS, Padova, Italy

**Keywords:** tumor microenvironment, colon rectal cancer, rhabdomyosarcoma, extracellular matrix, microenvironment remodeling

## Abstract

Cells and extracellular matrix (ECM) components represent the multifaceted and dynamic environment that distinguishes each organ. Cancer is characterized by the dysregulation of the composition and structure of the tissues, giving rise to the tumor milieu. In this review, we focus on the microenvironmental analysis of colorectal cancer (CRC) and rhabdomyosarcoma (RMS), two different solid tumors. While a lot is known about CRC environment, for RMS, this aspect is mostly unexplored. Following the example of the more complete CRC microenvironmental characterization, we collected and organized data on RMS for a better awareness of how tissue remodeling affects disease progression.

## Introduction

For many years, the cellular compartment has been considered the main target both to classify the disease and to develop a therapy. However, in the past two decades, the role of the non-cellular part, the extracellular matrix (ECM), has been taken into consideration as a key element that, together with cells, contributes in TME building (1).

Fibroblasts are the main players in ECM remodeling. In physiological conditions, they remain quiescent, but in wound healing response, they can transiently activate to myofibroblasts and participate in synthesis and deposition of ECM proteins (2). The activated fibroblasts in the tumor microenvironment have different features compared to myofibroblasts and are called cancer-associated fibroblasts (CAFs). CAFs in tumor stroma are stably active, and they have enhanced migratory capacity and secrete pro-tumorigenic growth factors and chemokines (3).

Immune cells in tumor microenvironment have a crucial role not only in malignant cell recognition and suppression but also in immunological escape. Macrophages can be polarized in two distinct phenotypes: anti-tumorigenic M1 producing reactive oxygen species and inflammatory cytokines, or pro-tumorigenic M2. Macrophages residing in an immunosuppressive tumor environment are usually shifted to a pro-tumorigenic subtype (M2) (4, 5). Tumor-infiltrating lymphocytes (TILs) can differentiate into many other different subtypes: CTLs, Th1, Th2, Th17, and Treg; among them, Tregs exert pro-tumorigenic activity. Myeloid-derived suppressor cells (MDSCs) are recruited in the tumor stroma from the bone marrow, where they mediate immunosuppressive functions as recruitment of Tregs (6–8).

New blood vessel formation is crucial for nutrient supply, waste removal, and metastatic dissemination: endothelial cells (ECs), together with pericytes, are responsible for angiogenesis and supporting tumor growth. Angiogenesis can be triggered not only by cancer cells in a hypoxic environment but also by stromal and infiltrating immune cells. Among the factors that promote angiogenesis, the hypoxia-inducible factor (HIF), the vascular endothelial growth factor (VEGF), and the platelet-derived growth factor (PDGF) play a key part (9). The role in tumor vascularization of pericytes is not completely understood, evidences underline a modulation of EC permeability, vessel stabilization, and metastasis together with degradation of endothelial ECM (10).

Collagens, proteoglycans and glycoproteins (laminins, elastin, fibronectin) are the main components of ECM. The ECM is a dynamic scaffold that provides structural organization and physical support to cells within the tissue, mediating also the release of growth factors and signaling molecules in a time- and context-dependent manner. Abnormal microenvironment can dysregulate the behavior of stromal cells in the TME and facilitate angiogenesis, inflammation, and tumor growth (11, 12).

In the last decade, studies on the microenvironment of the epithelial cancers, such as breast, lung, and colon tumors, with respect to sarcoma have been particularly developed. Indeed, the availability of samples together with the well-defined elements that constitute the microenvironment of the epithelial tumors allowed the achievement of a quite deep knowledge of the milieu with which cells interact (13–15). Consequently, the study of the altered, pro-tumorigenic environment has been considered as a new therapeutic approach to eradicate the disease. On the other hand, the sarcoma tumors are a quite heterogeneous group of mesenchymal malignancies (16). Patient-derived samples are mostly not available for research use, mainly because (i) sarcoma are rare and (ii) the small samples are devoted to pathologists and geneticists. For all these reasons, we thought that the data collection on what is the present knowledge on carcinoma vs. sarcoma microenvironment could be of interest for the scientific community. With both carcinoma and sarcoma occupying a wide field in cancer studies, we decided to start from our expertise, and for this minireview, we chose to consider colorectal cancer (CRC) and rhabdomyosarcoma (RMS) as peculiar examples of carcinoma and sarcoma, respectively.

In this manuscript, we will focus on the microenvironment of CRC and RMS in order to draw the attention from a cell-centric description on the malignancies to a more complete overview on the TME. To underline the importance of microenvironment, a quite recent classification according to milieu composition has been proposed for CRC (17). A clearer understanding of TME elements, such as immune and stromal cells can help stratification of patients and the design of new approaches for personalized medicine (18). Tables 1, 2 summarize the CRC and RMS TME players.

**Table 1:** Summary of the cell types in CRC and RMS microenvironment.

	**Markers**	**Cells**	**Abbreviation**	**CRC**	**RMS**	**References**
**CELL TYPES OF THE MICROENVIRONMENT**						
Immune cells	CD3+ CD4+	T helper lymphocytes	Th1/Th2	+	±	(19–21)
	CD3+ CD8+	Cytotoxic T lymphocytes	TCL	+	±	(19–21)
	CD4+ CD25+ Foxp3+	T regulatory lymphocytes	Treg	+	+	(6, 22)
	CD68+ CD163+ CD14+	M2 macrophage	M2	+	+	(13, 21, 23–27)
	CD68+ CD80+ CD86+	M1 macrophage	M1	+	n.f.	(13, 23, 25)
	CD3+ CD56+	Natural killer T lymphocyte	NKT	+	+	(24, 28)
	CD3– CD56+	Natural killer	NK	+	+	(24, 28)
	IgM CD19 CD20	B lymphocytes		n.f.	–	(18, 24)
Vasculature	CD31+ CD34+ VEGFR-2+ factor VIII+ vWF +	Endothelial cells		+	+	(10, 29)
	α -SMA Desmin CD146+ NG2+	Pericytes		+	+	(10, 30, 31)
	CD31– PAS+ Lam+	Vasculogenic mimicry tumor cells		n.f.	+	(32–34)
Fibroblast	VIM+ DES+ FSP1+	Quiescent fibroblast		n.f.	–	(29, 35)
	α-SMA+ PDGFRα+ PDGFRβ+ FAP+	Activated fibroblast/myofibroblast		n.f.	–	(29, 35)
	α-SMA+ FAP+ TNC+ NG2+	Cancer-associated fibroblast	CAF	n.f.	n.f.	(35–37)

*n.f., not found*.

**Table 2:** Summary of the ECM–cell interaction molecules.

	**Molecules**	**Function**	**CRC**	**RMS**	**References**
**Extracellular matrix and matrix-associated molecules**					
Integrins	α6β4	Promote tumor cell migration	+	n.f.	(38)
Structural proteins	Fibronectin	Promote invasion	+	+	(39, 40)
	Collagen IV, V	Chemotherapy resistance?	n.f.	+	(40, 41)
	Laminin	Reduced adhesion to laminin enhances cell migration	+/−	+	(13, 38, 40, 42)
Proteoglycans	Syndecan-1	EMT	+	n.f.	(13, 43)
	Hyaluronic acid	Proliferation and mobility	+	n.f.	(13, 43)
Metalloproteases (MMPs)	MMP-1	Remodeling of ECM. ARMS aggressiveness	–	+	(44)
	MMP-2	Remodeling of ECM. ARMS aggressiveness	–	+	(44)
	MMP3	Low levels of microsatellite instability	+	–	(45)
	MMP-9	Remodeling of ECM. ARMS aggressiveness	–	+	(44)
	MMP12	Remodeling. Good prognosis	+	–	(45)
	TIMP-1 (metalloprotease 1 inhibitor)	Resistance to chemotherapy	+	–	(44, 46, 47)

## Colorectal Carcinoma

Worldwide, CRC is the fourth most common cancer in both sexes and one of the major causes of mortality (48). The survival of CRC patients is highly correlated to the disease stage at diagnosis (49). CRC etiology is characterized by acquisition and progressive accumulation of genetic and epigenetic mutations by the somatic cells that confer a malignant phenotype.

However, only recently has the cell-centric view of tumor been replaced by a broader view that includes the complex TME in which the tumor cell grows, subsists, and co-evolves with it (50, 51).

### Cellular Components of CRC

#### Cancer-Associated Fibroblast (CAF)

Fibroblasts are the major stromal population of healthy colon mucosa; following neoplastic transformation of the epithelial cells, fibroblasts become the major component of the tumor stroma and contribute to the maintenance of this malign state through a molecular mechanism based on an “efferent” and an “afferent” pathway (52). In the “efferent” pathway, cancer cells secrete soluble signals responsible for fibroblast differentiation into CAF (36, 53). On the other hand, CAFs along the “afferent” pathway affect tumor cells proliferation, migration, and invasion (30, 54). CAFs are the most abundant stromal population in CMS4 CRC subtype, suggesting a possible involvement in building highly vascularized and inflammatory tumors. In a study of Herrera and colleagues, it was demonstrated that co-cultivating CRC cell line with CAFs increases CRC cell line migration potential. In addition, they proposed a CAF gene expression profile and a CAF signature that clusters patients with CRC into high- and low-risk groups (55). In a further study, Berdiel-Acer et al. derived the transcription profile of healthy fibroblasts, CAFs from primary CRC, and CAFs from CRC liver metastasis. Finally, they obtained a 19-gene signature, able to predict tumor recurrence in two independent datasets (56).

#### Immune Cells

Since the second decade of the 1900s, the hypothesis that immune cells represent a host defense mechanism against cancer and that the abundance of immune infiltrates correlated with patients survival was formed (57). In particular, for CRC, the first evidence supporting this hypothesis was published at the end of the 1970s (57–59). Further studies led to the identification of the most important players in this complex mechanism; this category of cells acquired the name tumor-infiltrating lymphocyte (TIL), which is composed of T-lymphocytes, B-lymphocytes, macrophages, and natural killer cells (19, 22).

A study proposed by Suzuki et al. in CRC liver metastasis patients demonstrated that high peritumoral mast cell (MC) infiltration, positive to tryptase, predicts poor prognosis (60). A CRC with high density of MCs has been related to tumor aggressiveness and reduced survival. Finally, increased peritumoral infiltrating MCs have been demonstrated as a predictive marker of poor outcome in CRC liver metastasis patients.

Recently, it has been demonstrated that tumor-associated neutrophils (TANs) can be polarized by the tumor microenvironment into cells with an N1 or N2 phenotype. These phenotypical and molecular-different cells have anti-tumor or pro-tumor properties, respectively. The anti-tumor TAN-N1 actively expresses immunoactive cytokines and chemokines and decreased arginase expression (61). By contrast, the pro-tumoral TAN-N2 decreased the expression of inflammatory-promoting molecules and increased arginase expression, causing the inactivation of T cell effectors (13). Interestingly, in advanced CRC, the extent of the systemic immune response, revealed by the lymphocyte/neutrophils ratio, has been correlated to a poor outcome (62, 63).

Stromal cell subtypes that are predominant in CRC microenvironment are the tumor-associated macrophages (TAMs). In CRC, longer patient survival and metastasis absence are associated with anti-tumoral TAM infiltration. Conversely, poor prognosis and cancer progression are associated with pro-tumoral TAM infiltration (23). However, despite this rigid classification based on phenotypic and molecular characteristics, it is still controversial if M1 or M2 macrophages strictly exert an anti- or pro-tumor activity in CRC TME. In fact, the modulation of their activity seems to be localized within the tumor mass. TAMs at the tumor edges induce apoptosis in cancer cells, while TAMs localizing at the invasive front showed a resistant phenotype to suppressive TME (64, 65). Recent studies highlight that TAM plasticity, modulated by different microenvironmental factors, has a prognostic impact on CRC patients (64, 65). An integrative study, proposed by Mlecnik et al. showed that among the heterogeneous group of CRC patients with a mismatch repair-deficient and microsatellite instability-high (MSI-H) phenotype, two groups can be distinguished: the most represented group with high T-cell activity and improved prognosis, and a minor group with reduced T-cell activity and poor prognosis (66). Recent studies showed that the evaluation and characterization of TIL at the tumor site could be used also as predictive factor for the response to immunotherapy. In this context, a preliminary study proposed by Le et al. demonstrated that mismatch repair-deficient and hypermutated MSI-H metastatic CRC patients could significantly benefit from treatment with anti-PD-1 (67). After the seminal observation, they demonstrated that mismatch repair and microsatellite instability status were able to predict clinical benefits to anti-PD1 treatment in metastatic CRC patients (18). In a recent study, Overman et al. observed that single-immunotherapy treatment using anti-PD1 was more effective in the MSI-H metastatic CRC group compared with the non-MSI group (68). A similar result was reported with the double-immunotherapy treatment combining anti-PD1 with anti-CTLA4 (68).

#### Endothelial Cells

Angiogenesis in CRC is finely orchestrated by a series of vascular factors such as VEGF family proteins and receptors, placental growth factor (PLGF), and two neuropilin co-receptors (NRP1 and NRP2) (69, 70). At the CRC invasion front, a high vascular density correlates with tumor recurrence and metastasis (10). The increased expression of VEGFR-2 in CRC liver metastasis promotes the neo-vascular switch, increasing the nutrient supply fundamental for tumor progression (69). Furthermore, PlGF/VEGFR-1 signaling promotes CRC invasion through a p38-MMP9 pathway and is associated with a worse prognosis (71). CRC patients often present at systemic level tumor-derived ECs, expressing both epithelial and mesenchymal markers (72).

Finally, in a recent study, Lu et al. proposed ECs not only as a vessel-forming cells but they also demonstrated that ECs were able to activate the Notch signaling pathway in CRC cells through a paracrine secretion of Jagged-1, increasing their stemness and chemoresistance (73).

### Acellular Components of CRC: ECM and Matrix-Associated Molecules

In order to progress and invade, cancer cells must overcome the physical barriers by interacting with ECM and its components. A study of Rabinovitz et al. demonstrated that in CRC cell lines, α6β4 integrin promotes cell migration by interacting with laminin-1 (38). Zapatka et al. demonstrated that the inactivation of tumor suppressor Smad4 gene, a late event on the carcinogenic chain of CRC, leads to a decreased expression of all three genes encoding laminin-5 that can favor invasion and metastasis spread (74). Ding et al. demonstrated that fibronectin, a high-molecular-weight adhesive glycoprotein present in the ECM, can promote invasion of colon cells via an up-regulation of focal adhesion kinase (39). Proteoglycans are a subset of molecules altered in CRC. Hashimoto et al., showed that basolateral border expression of syndecan-1 is associated with normal colonic epithelial cells, while in CRC, its expression is completely lost and this has been correlated with malignancy, epithelial–mesenchymal transition, tumor-node-metastasis stage, and local lymph node metastasis (43). Glycosaminoglycans are another key ECM component frequently deregulated in cancer. In CRC, hyaluronic acid (HA) enhances cell proliferation and motility *in vitro* and *in vivo* (42, 75). Dunn et al. showed that the inhibition of HA production in SW620 blocks *in vitro* Matrigel invasion (76). In HCT-116 cells, the interaction of HA with CD44 stimulates cell survival, proliferation, adhesion, and invasion through ERBB2 activation (77, 78).

The proteinases that regulate ECM turnover and remodeling are another intriguing component of ECM. Zucker et al. demonstrated that matrix metalloproteases (MMPs) are correlated with tumor stage and prognosis. In this context, the MMPE up-regulation correlates with MSI-L and bad prognosis. Conversely, overexpression of MMP12 is associated with a better prognosis in CRC (45). Davidsen et al. demonstrated that CRC cells actively expressing TIMP-1 protein showed an increased resistance to drugs compared to TIMP-1 silenced cells (46). In line with this study, Sorensen et al. showed that high TIMP-1 level in CRC tissue and plasma correlated with a bad prognosis (47).

## Rhabdomyosarcoma

Among the tumors of mesenchymal origin, RMS is the most common soft tissue sarcoma in children and young adults with an incidence of 4.5 cases among 1,000,000 newborns. The two main subtypes are the embryonal rhabdomyosarcoma (ERMS) and alveolar rhabdomyosarcoma (ARMS), accounting, respectively, for the 57% and the 23% of all diagnosed RMS (79). ERMS is associated with a better prognosis and higher relative 5-year survival rates (73.4%). ARMS is associated with poorer outcome and a lower 5-year survival rate (47.8%) due to the high aggressiveness and tendency to metastasize (79, 80). Following the guidelines of the European Pediatric Soft Tissue Sarcoma Study Group (EpSGG) for RMS 2005 protocol, patients diagnosed with RMS were stratified in four risk groups: low, standard, high, and very high risk. Prognostic factors considered are: pathology (favorable for embryonal, spindle cells and botryoid RMS and unfavorable for ARMS), post-surgical stage (from complete resection to macroscopic residual), site of onset, lymph node involvement, size of the mass, and age of the patient (81). Similarly, the guidelines for RMS patient stratification given by the Children's Oncology Group identify four risk categories (low risk subset 1, low risk subset 2, intermediate risk, and high risk) considering histology, site of onset, size, nodal involvement, presence of distant metastases, and Intergroup Rhabdomyosarcoma Study classification based on residual disease after surgery (82). In both protocols, stromal cell population and the TME are not considered for diagnostic purposes.

### Cellular Components of RMS

#### Cancer-Associated Fibroblast (CAF)

The role of fibroblasts in RMS has not been precisely investigated yet. RMS cell lines express Macrophage migration Inhibitory Factor (MIF). An interesting result obtained by Tarnowski and colleagues demonstrate that MIF, interacting with RMS cell surface receptors CXCR4 and CXCR7 in a paracrine loop, increases cell adhesion, vascularization, and reduces the number of infiltrating CAF. Down-regulation of MIF in the RMS cell line, used for xenograft production, resulted in bigger sized xenografts, higher stromal cell support, and a higher number of circulating tumor cells (37). The presence of a stromal compartment in sarcomas has been questioned in the study of Tomlinson et al., where the difference in the pattern of blood vessels distribution in sarcoma and carcinoma tumor masses has been attributed to the presence of fibroblasts and myofibroblasts in the latter, and the absence of these cells in the former (29).

#### Immune Cells

The presence of the immune compartment in RMS is still debated. D'Angelo and colleagues selected a cohort of 50 patients with soft tissue sarcomas to examine the immune milieu. CD3+ (TILs), CD4+ (T-helper cells), CD8+ (cytotoxic T-cells), and FOXP3+ (Treg) lymphocytes were found in 98% of the biopsies, while macrophages were found in 90% of the cases. The lower presence of CD3+− and CD4+− infiltrating lymphocytes correlates with a favorable outcome (20), in contrast with a larger dataset of different tumors showing a positive correlation between CD3+ and CD4+ infiltrates and survival (83). Higher number of CD8+ cells were found in patients with larger tumors or with metastasis (20). However, this study presents some critical limitations: the low number of tumor specimens representing each histological subtype (20 different subtypes represented by 1 or 2 specimens each) and samples representing the same malignancy but with different stages of the disease. A recent work divided a cohort of 25 RMS (13 embryonal, 11 alveolar, and 1 sclerosing) into 4 categories based on the expression of PD-L1. Although RMS cells were PD-L1^−^, immune infiltrating cells (CD3+ lymphocytes and CD68+ macrophages) were found in 6/11 and 9/14 of ARMS and ERMS specimens, respectively, with different patterns and grades of PD-L1 positivity. PD-L1-positive immune cells were observed within the tumor burden in 4/25 of the cases, in the surrounding stroma in 5/25 of the cases, and finally in 7/25 of the specimens, very few T-cells in either the parenchyma or the stroma could be found. However, due to the limited size of the cohort, authors could not identify any correlation between PD-L1 status and RMS subtypes or the clinical outcome (24). Regarding the relation between RMS cells and the immune system *in vitro*, it is shown that cytotoxic drugs such as doxorubicin provoke the translocation of calreticulin from the endoplasmic reticulum to the cell surface and, in combination with anti-PD-L1 antibody, increase the efficiency of phagocytosis by macrophages (84). Moreover, doxorubicin increases MIF expression in RMS cell lines. MIF induces the differentiation of pro-tumorigenic CD33+ CD14+ myeloid-derived suppressor cells (MDSCs) from peripheral blood mononuclear cells (PBMCs). However, inhibition of MIF impairs the migration potential of RMS cells (85), in contrast with the results obtained in the work previously mentioned (37).

#### Endothelial Cells

The presence of ECs and evidences of vascularization in soft tissue sarcomas (STS) were investigated by West and colleagues who evaluated the microvessel density (MVD) in 42 high-grade STS patients with different histology. Diffuse staining of VEGF was reported in 41/42 samples but without any correlation with microvessel density. Hot spots of angiogenesis were found in only 33% of the tissue specimens, highlighting a homogeneous microvessel distribution that, however, did not correlate with survival of the patients or presence of metastasis (86). The main signals promoting angiogenesis in RMS are hypoxia, VEGF (87), and PDGF (88, 89). To stimulate angiogenesis, VEGF, expressed in all the cell lines tested, and its receptors, in particular VEGFR-1 that is present in 4/6 of the cell lines considered, sustain an autocrine positive feedback-loop in RMS cell lines, promoting proliferation, and cell growth (90). VEGF is expressed more frequently in ARMS (70.6%) than in ERMS (50.0%) and its expression is associated to poor prognosis for both subtypes, representing a valuable potential therapeutic target (87). An alternative, or complementary mechanism for blood diffusion within the tumor is the vasculogenic mimicry (VM): some tumor cells express endothelium-associated genes and form loops and arc networks rich in laminin lined with other tumor cells. These “channel-like spaces” provide a blood perfusion and a dissemination route (32). Two different studies highlight the vasculogenic mimicry mechanism in sarcomas: the first consider a cohort of 32 patients with orbital RMS, showing a strong correlation between VM and poor outcome (33). The latter analyzes a larger cohort of patients showing positive correlation between VM and poorer outcome in mesothelial sarcoma and ARMS patients (34).

### Acellular Components of RMS: ECM and Matrix-Associated Molecules

The characterization of RMS ECM itself is still incomplete. Scarpa and colleagues, in 1987, studied *in vitro* the matrix composition of different pediatric tumor cell lines, and they reported high variability between RMS subtype considered (alveolar, embryonal, unclassified, and synovial sarcoma). ARMS and ERMS produce different matrices: the former synthetize mainly collagen V and laminin and the latter almost exclusively collagen IV and fibronectin. In both cases, deposition of collagen I and III, interstitial collagens typical of mesenchymal cells, was not reported (40). Interactions between ECM and RMS cells are also altered: the expression of α-dystroglican, important complex for laminin and basement membrane assembly and binding, is down-regulated in RMS and other pediatric solid tumors. Even if the implication of this differential expression in tumor biology has yet to be clarified, reduced adhesion to laminin of these cells can result in enhanced migration ability (91). The matrix degradation in the tumor environment is fundamental for tumor development and invasiveness, releasing growth factors, guiding cell migration, and promoting angiogenesis. Of particular interest is that remodeling enzymes MMP-1, MMP-2, and MMP-9 are found to be up-regulated in ARMS compared to ERMS, and this can be one of the factors responsible for the higher aggressiveness of the alveolar subtype (44).

## Concluding Remarks

Drawing the attention toward the microenvironment, the epithelial origin of carcinoma characterizes the development of a microenvironment with common cellular and non-cellular elements that can be found in colon, lung, and breast (13–15). On the other hand, sarcomas are rare cancers of mesenchymal origin and are characterized by high heterogeneity of the histologies and genetic and clinical features. In the past, sarcoma and, in particular, RMS were believed “one-compartment tumors” with mainly cancer cells (29). Now it is more clear that, although at a lower level with respect to carcinoma, the stromal cell heterogeneity is present and it is worth studying it (92). Indeed, the multifaceted nature of the TME has an impact on tumor progression, as both tumor and stromal cells change during time, developing angiogenic, invasive, and migratory properties. Common targets for therapeutic approaches of CRC and RMS are focused on antiangiogenic or immunomodulating effects, although the still poor knowledge of RMS TME makes this aspect difficult to develop. While significant progresses have been made for the study of TME in CRC, to date, precise characterization of the tumor stroma for soft tissue sarcomas, and particularly for RMS, is still lacking and urgently needed. It is conceivable that the cancer cells that survived also after chemotherapy are stem cells protected by the surrounding microenvironment where the stem cell niche is still intact. In this perspective, a deeper characterization of the microenvironment, and specifically for RMS, will help to identify (i) the mechanisms of tumor growth, development, and metastasis; (ii) how the crosstalk between cells in the stroma participate to the tumor progression; and finally (iii) new microenvironment-related therapeutic targets. These, in combination with present therapies, could open new avenues to ameliorate the overall survival of the patients.

## Author Contributions

MS and ED'A wrote the article. GB and MA supervised the work. MP wrote the article, supervised, and gave the final approval.

### Conflict of Interest

The authors declare that the research was conducted in the absence of any commercial or financial relationships that could be construed as a potential conflict of interest.
